# Diversity and function of OXA-48-like β-lactamase variants in environmental *Shewanella* isolates from Stockholm, Sweden

**DOI:** 10.1128/aem.00125-26

**Published:** 2026-05-21

**Authors:** Víctor Fernández-Juárez, Marija Petrovic, I. Mihindukulasooriya, Enrique Joffré, Åsa Sjöling, Alberto J. Martín-Rodríguez

**Affiliations:** 1 Department of Microbiology, Tumor and Cell Biology, Karolinska Institutet 310388, Stockholm, Sweden; 2 Department of Medical Biochemistry and Microbiology, Uppsala University 211745, Uppsala, Sweden; 3 Department of Chemistry and Molecular Biology, University of Gothenburg 191648 , Gothenburg, Sweden; 4 Department of Clinical Sciences, University of Las Palmas de Gran Canaria 16750, Las Palmas de Gran Canaria, Spain; Centers for Disease Control and Prevention, Atlanta, Georgia, USA

**Keywords:** beta-lactamase, *Shewanella baltica*, nitrocefin hydrolysis, antimicrobial resistance, environmental reservoirs

## Abstract

The genus *Shewanella* is a recognized reservoir of antibiotic resistance genes (ARGs), including chromosomally encoded *bla*_OXA_ alleles that have given rise to clinically relevant OXA-48-like β-lactamases in *Enterobacterales*. However, our understanding of these enzymes in environmental *Shewanella* populations remains limited. Here, we investigated their distribution, evolution, and function in *Shewanella* spp. isolated from Baltic Sea environments near Stockholm, Sweden. Whole-genome sequencing of 25 isolates affiliated with *Shewanella baltica* and related genospecies revealed that each carried a chromosomal OXA-48-like β-lactamase closely related to OXA-551. These enzymes exhibited substantial N-terminal polymorphisms, including indels, defining 20 novel variants. Phylogenetic analyses showed that the diversification of OXA variants closely mirrored the host species taxonomy, suggesting parallel evolution. Phenotypic susceptibility testing demonstrated that native *Shewanella* hosts remained largely susceptible to carbapenems, third-generation cephalosporins, and, to a lesser extent, ampicillin, suggesting limited expression or activity under native regulatory control. Functional assays using Δ*bla*_OXA_ mutants of *S. baltica* strains carrying divergent variants revealed variable contributions to β-lactam resistance. In contrast, heterologous expression of these enzymes in *Escherichia coli* conferred high resistance to ampicillin and β-lactamase activity comparable to the reference OXA-551, as demonstrated by nitrocefin hydrolysis kinetics. Comparative analysis of *S. baltica*-like *bla*_OXA-48_ promoters and those associated with plasmid-borne OXA-48 variants in *E. coli* revealed conserved sigma70-dependent regulation, besides additional predicted transcription factor binding sites clustered near the −10 box, suggestive of a fine-tuned regulation in *Shewanella*. Our findings expand functional insights into OXA-48-like β-lactamases and highlight environmental *Shewanella* as reservoirs of OXA-48-like diversity.

**IMPORTANCE:**

Antimicrobial resistance (AMR) is a major global health challenge, with β-lactamase-mediated resistance undermining the efficacy of last-line antibiotics, such as carbapenems. OXA-48-like carbapenemases, now endemic across various parts of the world, trace their origin to *Shewanella* species. Understanding how these enzymes diversify, function, and transition from chromosomal genes to transmittable, clinically concerning resistance determinants is critical for AMR surveillance and risk assessment. This study demonstrates that Baltic Sea *Shewanella baltica* populations harbor diverse OXA-48-like enzymes with relatively limited phenotypic impact in their native hosts, but more active when expressed in *E. coli* mimicking real-life acquisition. By linking natural sequence variation to enzyme activity, we show that polymorphisms in the N-terminal region of these enzymes do not have substantial functional consequences, indicating that many naturally occurring variants reflect evolutionary mutations that have not affected enzyme performance. These findings reinforce the importance of aquatic environments as reservoirs of AMR determinants poised for mobilization.

## INTRODUCTION

The genus *Shewanella* is widespread in aquatic and sediment-associated environments worldwide, with one species, *Shewanella algae*, emerging as a human pathogen of increasing clinical relevance (1). *Shewanella* spp. are progenitors of several subclasses of Ambler class D β-lactamases (2–4), as well as other antibiotic resistance genes (ARGs) of clinical concern, such as *qnrA*, found in *S. algae* and closely related species (5), conferring resistance to fluoroquinolones, and *mcr-4.3*, thought to have originated from *Shewanella frigidimarina* (6), which mediates resistance towards colistin, a last resort antibiotic against infections caused by multidrug-resistant Gram-negative bacteria. All these chromosomally encoded *Shewanella* genes have been identified in an array of mobile genetic elements (MGEs), including plasmids, that are currently widespread among clinically important human pathogens (7–10).

Diverse ARGs occur naturally in environmental bacteria and can be mobilized into conjugative plasmids through recombination and subsequent dissemination via horizontal gene transfer (11). This process requires either the coexistence of native (primarily environmental) and non-native (primarily human-associated) bacterial hosts for sufficient periods of time, or efficient transmission routes across distant hosts, that is, from environmental bacterial communities to human pathogens, which are still poorly delineated. Not infrequently, ARG carriage is not associated with phenotypic resistance in the native bacterial host, which is typically attributed to low expression levels of the gene in question, less functional gene products, or various genetic factors (12, 13). These so-called “silent” genes may nonetheless effectively contribute to resistance when expressed in non-native hosts (12, 13).

Class D β-lactamases, also known as oxacillinases (OXAs) due to their substrate preference, are of particular concern due to their widespread occurrence in *Enterobacterale*s and their recent spread to high-risk, multidrug-resistant strains of *Pseudomonas aeruginosa* and *Acinetobacter baumanii* (14). Among them, OXA-48-like enzymes, the most widespread in *Shewanella* genomes, are known to contribute to carbapenem resistance and represent the preponderant carbapenemases across large areas of Europe, the Middle East, and Northern Africa (15). Within Europe, OXA-48 carbapenemases are so widespread in *Enterobacterales* that they are currently considered endemic in Belgium, France, and Spain (16). Currently, there are nearly 1,400 class D beta-lactamases registered in the Beta-Lactamase DataBase (BLDB) (17), including 68 OXA-48-like variants, which showcases the extensive sequence variability across this family of enzymes. Some *Shewanella*-native, chromosome-encoded OXA-48-like oxacillinases, such as OXA-54 in *Shewanella oneidensis* (18), OXA-55 in *S. algae* (19), or OXA-181 in *S. xiamenensis* (20), have been characterized biochemically and genetically. However, the evolutionary selective pressures shaping the diversity of OXA proteins and the impact of such diversity on enzyme function are still not well defined.

Our team has devoted efforts to the surveillance of antimicrobial resistance (AMR) and ARG dissemination in waterborne bacteria, including *Shewanella* populations closely related to *Shewanella baltica* collectively referred to as the *S. baltica* complex (21). Hence, this study was designed to (i) assess the diversity of class D β-lactamases in environmental *Shewanella* retrieved from Baltic Sea environments in the surroundings of Stockholm, Sweden; (ii) investigate the distribution of mutations in these enzymes in similar *Shewanella* hosts worldwide; (iii) evaluate the contribution of *bla*_OXA_ to AMR in native *Shewanella* hosts; and (iv) functionally characterize novel variants in a heterologous enterobacterial host, mimicking horizontal acquisition.

## MATERIALS AND METHODS

### Bacterial isolation

Water and sediment samples were collected from Nynäshamn (58°53′57.29″N, 17°57′2.23″E; May 14, 2022), Notholmen, Tyresö (59°13′58″N 18°18′40″E; May 14, 2022), and Hagaparken (59°21′21.0″N 18°02′37.0″E; April 11, 2022) representing brackish environments in the Baltic Sea (Nynäshamn and Notholmen) or Lake Brunnsviken (Hagaparken), connected to the Baltic Sea through the Ålkistan canal. *Shewanella* spp. were isolated from water samples upon filtration through 0.45-µm mixed cellulose ester membrane filters (Millipore) and plating on Lyngby’s Iron Agar (LIA) containing 0.04% (w/v) L-cysteine and either colistin (8 mg/L, due to the intrinsic resistance of some *Shewanella* sp.), or no antibiotic. For isolation from sediments, scooped shoreline sediment samples in 50-mL sterile centrifuge tubes were overlaid with sterile PBS, vigorously shaken, and serially 10-fold diluted before plating on colistin-supplemented or plain LIA. *Shewanella*-like colonies were identified as H_2_S producers in this medium after incubation at 28°C for 24 h, as previously described (21, 22), and purified by re-streaking on the same medium. Genus-level identification was achieved by matrix-assisted laser desorption/ionization-time of flight (MALDI-TOF) mass spectrometry (MS) analysis (MALDI Biotyper Sirius System, Bruker).

### Whole-genome sequencing and extraction of *bla*_OXA_ genes

*Shewanella* strains were grown overnight, and genomic DNA was extracted using the DNeasy Blood and Tissue kit (Qiagen) and sequenced, as previously described (5). Briefly, 50 ng of genomic DNA was used for library preparation with the MGIEasy FS Library Prep Set (MGI Tech). Equimolar pooled libraries were then circularized using the MGIEasy Circularization Kit (MGI Tech) and sequenced as 2 × 100 bp paired-end reads on an NBSEQ-G400 platform (MGI Tech). Following genome assembly and annotation with BACTPipe v3.1.0, annotated *bla*_OXA_ alleles were extracted.

### Phylogenetic analyses and bioinformatics

Sequence alignments were generated with ESPript 3.0 (23) using the sequence and structural data of OXA-48, available from the Protein Data Bank (PDB, entry 3HBR), as a reference. A maximum-likelihood phylogenetic analysis of OXA amino acid sequences was performed with MEGA X (24), using a Jones-Taylor-Thornton (JTT) matrix-based model with Gamma (G) distribution and 1,000 bootstrap iterations. DnaSP v6 (25) was used for the calculation of Tajima’s and Fu & Li’s statistics from DNA sequences.

A total of 106 *Shewanella baltica* genome sequences (combining the 25 from this study and those publicly available from the NCBI GenBank), 219 sequences from *Shewanella algae*, 32 from *S. oncorhynchi*, and 98 from *S. xiamenensis* (File S1) were aligned using the Clustal Omega tool (EMBL-EBI), in four different batches, respectively. The resulting multiple sequence alignments were used to generate a sequence logo via the WebLogo server (https://weblogo.berkeley.edu) and to analyze the distribution of amino acid residues across the alignment, as well as the degree of conservation at variable positions, according the Gonnet substitution matrix.

Protein structures and structure-based functional annotations of the OXA enzymes from strains N1WShe5-IV (OXA-1408), N1WShe6 (OXA-1410), T1SShe5-III (OXA-1413), and T1WShe4 (OXA-1417) were predicted using Iterative Threading ASSEmbly Refinement (I-TASSER) (26), and the resulting models were visualized and aligned with the *Klebsiella pneumoniae* OXA-48 reference structure (3HBR) in Pymol v3.0 (Schrödinger, LLC).

Putative promoters and transcription factor binding sites were predicted with BPROM (https://www.softberry.com/) and visualized using a custom Python script. Synteny plots were generated with the Clinker pipeline (27).

### Strains and growth conditions

*Escherichia coli* TOP10 was grown in Miller’s LB medium at 37°C, supplemented with tetracycline (Tc, 15 µg/mL) when required. *Shewanella* sp. N1WShe5-IV, N1WShe6, T1SShe5-III, and T1WShe4, or isogenic Δ*bla*_OXA_ mutant derivatives, were routinely cultured in Miller’s LB medium at 28°C. *E. coli* MFDpir was grown on LB agar supplemented with diaminopimelic acid (0.3 mM) and, when required, Tc, at 37°C, except during conjugal mating with *Shewanella*, which was conducted at 28°C. The strains employed in this study are presented in Table S1.

### Genetic manipulations

To express distinct OXA variants from a promoterless vector, we generated a custom pGEN-MCS derivative (28), named pGEN-MCS-Tc, in which the *bla* selection cassette and its promoter were replaced by a *tet* cassette and its promoter (obtained from plasmid pSIM27 [29]) using the XbaI and SpeI restriction sites (Table S2). Divergent *bla*_OXA-551_-like alleles of strains N1WShe5-IV, N1WShe6, T1SShe5-III, and T1WShe4 were cloned into plasmid pGEN-MCS-Tc using the primers indicated in Table S2, which spanned the gene coding sequence and the upstream intergenic region containing the putative promoter. The reference *bla*_OXA-551_ sequence in the Comprehensive Antibiotic Resistance Database (CARD) (Accession ARO:3005775) and its native promoter, obtained from *Shewanella* sp. VAX-SP0-4CM-5 (30), were cloned into the same sites. In addition, the previously characterized OXA-54 from *S. oneidensis* MR-1 was cloned into pGEN-MCS-Tc following the same strategy and used as a reference in subsequent studies. All constructs were verified by Sanger sequencing. The recombinant plasmids and the empty vector were mobilized into *E. coli* TOP10 by chemical transformation.

The *bla*_OXA_ gene was deleted from strains *Shewanella* sp. N1WShe5-IV, T1SShe5-III, and T1WShe4 by allelic replacement following described procedures (31). In brief, the upstream (585 bp) and downstream (600 bp) regions flanking the gene coding sequence were sequentially cloned into plasmid pKNG101 using the primers listed in Table S2. The suicide vector was propagated in *E. coli* DH5α λpir, mobilized into calcium-competent *E. coli* MFDpir, and transferred to each *Shewanella* strain by biparental mating. Merodiploids were resolved by plating on LB agar supplemented with 10% sucrose (w/v). Gene deletions were confirmed by gel electrophoresis with PCR primers flanking the recombination sites (Table S2).

### Antibiotic susceptibility testing

The susceptibility of environmental *Shewanella* isolates and *E. coli* TOP10 strains expressing reference or divergent OXA variants, or carrying the empty cloning plasmid, toward ampicillin (AMP, 10 µg), ceftazidime (CAZ, 10 µg), cefotaxime (CTX, 30 µg), imipenem (IMP, 10 µg), and meropenem (MEM, 10 µg) was determined by the disc diffusion method. For *Shewanella*, the medium was supplemented with 0.5% NaCl (w/v), due to the comparatively poor growth observed for these strains on plain Mueller Hinton medium, and the incubation temperature was 28°C, instead of 35°C, as the latter was found to be nearly non-permissive for the growth of many of the retrieved strains. The minimal inhibitory concentration (MIC) of the same antibiotics to *E. coli* TOP10 strains was additionally tested by the broth microdilution method, and interpreted following EUCAST guidelines (32).

### Kinetic assays of nitrocefin hydrolysis

*E. coli* TOP10 strains carrying the recombinant *bla*_OXA_ expression plasmids or the empty vector control were pre-grown in LB broth with Tc overnight at 37°C. Overnight cultures were diluted 1:100 in 20 mL of fresh LB medium without antibiotic and grown to an OD_600_ of 0.58 ± 0.02. Cell pellets were then obtained by centrifugation at 3,273 × *g*, 4°C, and stored frozen at −80°C for 24 h. Cell lysis was completed by addition of 2 mL PBS (0.01 M, pH 7.4) to the thawed pellets and sonication on ice (8 × 10 s, with 30-s resting periods between sonication steps, amplitude 50%). Cell debris was removed from the whole-cell lysates by centrifugation at 16,000 × *g*, 4°C, 20 min. Upon quantification of total protein content by the BCA method (33), equal amounts of protein (10 µL) were mixed, in duplicates, with 90 µL of nitrocefin solution in PBS (200 µM, final concentration) inside the wells of a 96-well plate. Nitrocefin hydrolysis was simultaneously monitored by recording the OD_490_ (increase proportional to the generation of its hydrolytic product) and the OD_380_ (decay proportional to the loss of intact nitrocefin) every 2 min for 2 h.

Additional substrates, including CENTA (a chromogenic β-lactamase substrate) and imipenem (a carbapenem), were evaluated to further assess enzymatic activity of novel and reference OXA enzymes. For CENTA, assays were performed using cell lysates prepared as described above, with a final substrate concentration of 800 µM. Hydrolysis was followed for 1 h by monitoring OD_405_ increase (34). Hydrolysis of imipenem was assessed using the Blue-Carba method (35). Briefly, this assay consisted of an aqueous solution of bromothymol blue (0.04% w/v) supplemented with imipenem (3 mg/mL) with a final pH of 7.0 (35). A loopful of bacterial culture was inoculated into 100 µL of the reaction mixture and incubated with shaking for 2 h. A color change from blue to yellow was interpreted as a positive result, indicating imipenem hydrolysis. For both assays, a positive control was included, using an ESBL *E. coli* expressing CTX-M-55 (Martín-Rodríguez laboratory collection).

### Data representation and statistical analysis

GraphPad Prism v10.0.2 was employed for data visualization and statistics, unless otherwise indicated.

## RESULTS

### Baltic Sea *Shewanella* isolates carry *bla*_OXA-48_-like variants with substantial N-terminal diversity

To investigate the diversity of OXA proteins in the local *Shewanella* populations, we extracted the *bla*_OXA_ coding sequences from 25 arbitrarily selected and whole genome sequenced *Shewanella* isolates retrieved from water and sediment samples collected in Nynäshamn, Tyresö, and Hagaparken, as representative members of the natural *Shewanella* communities in the Baltic Sea region surrounding Stockholm. The species affiliation of the isolates was *Shewanella baltica* or closely related genospecies. Each isolate harbored a single, chromosomal *bla*_OXA_ gene, encoding a class D OXA-48-like β-lactamase closely related to the OXA-551 subtype, which altogether represented 20 novel OXA-48-like variants, termed OXA-1401 to OXA-1418, OXA-1428, and OXA-1429.

**Fig 1:**
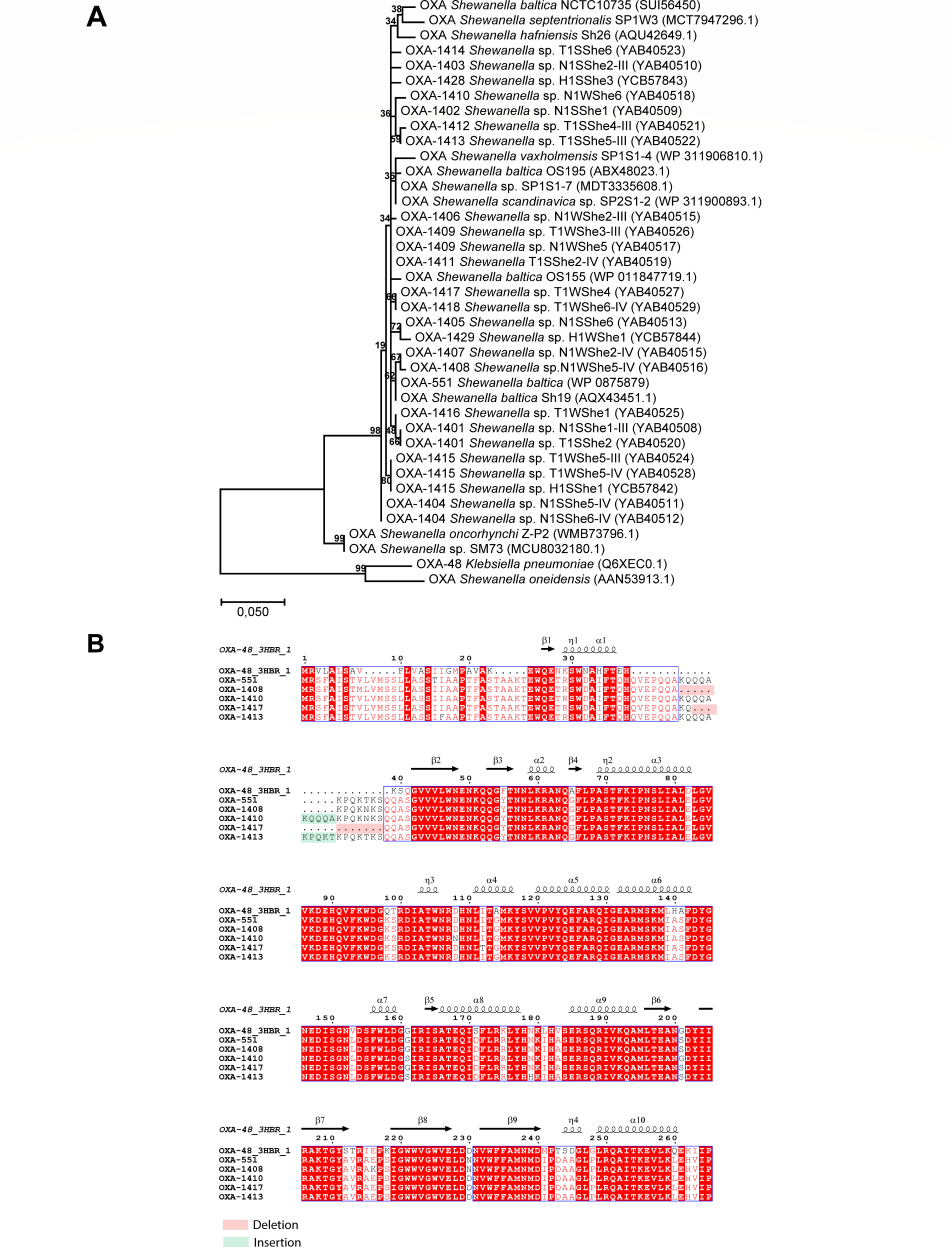
Diversity and phylogeny of OXA-48-like variants of *S. baltica* complex strains. (**A**) Phylogenetic reconstruction of precursor sequences of OXA enzymes extracted from the whole genome sequences of the *S. baltica* strains of this study and representative sequences from closely related and more distant *Shewanella* species, as well as the reference OXA-48 sequence. GenBank accessions of full-length protein sequences are given in parentheses. The maximum-likelihood evolutionary relationships were reconstructed using the JTT + G model after 1,000 bootstrap iterations. (**B**) Sequence alignment of OXA-48, OXA-551 (CARD accession ARO:3005775), and a novel variant carried by strain N1WShe5-IV (OXA-1408) presenting diverse polymorphisms with respect to OXA-551. Secondary structural elements are based on the crystal structure of OXA-48 (PDB entry 3HBR). Red and green shadows in the plot indicate deletions and insertions, respectively.

To delineate the evolutionary relationships of these *Shewanella* OXA β-lactamases within the broader *Shewanella* OXA-48-like family, a phylogenetic reconstruction was generated using the best-fit model (JTT + G) as implemented in MEGA X (Fig. 1A). This analysis evidenced well-supported associations that correlate with the taxonomic position of the harboring strains, with the OXA variants of *S. baltica* and closely related species, such as *Shewanella scandinavica*, *Shewanella vaxholmensis*, *Shewanella septentrionalis*, or *Shewanella hafniensis* clustering apart from those of other more distant species inhabiting similar environments, such as *Shewanella oncorhynchi* or *Shewanella oneidensis*, the latter closely related to the archetypal OXA-48 reference of *Klebsiella pneumoniae* used as an outgroup for the phylogenetic reconstruction (Fig. 1A). This evidence suggests evolution of OXA enzymes in *Shewanella* parallel to species diversification.

**Fig 2:**
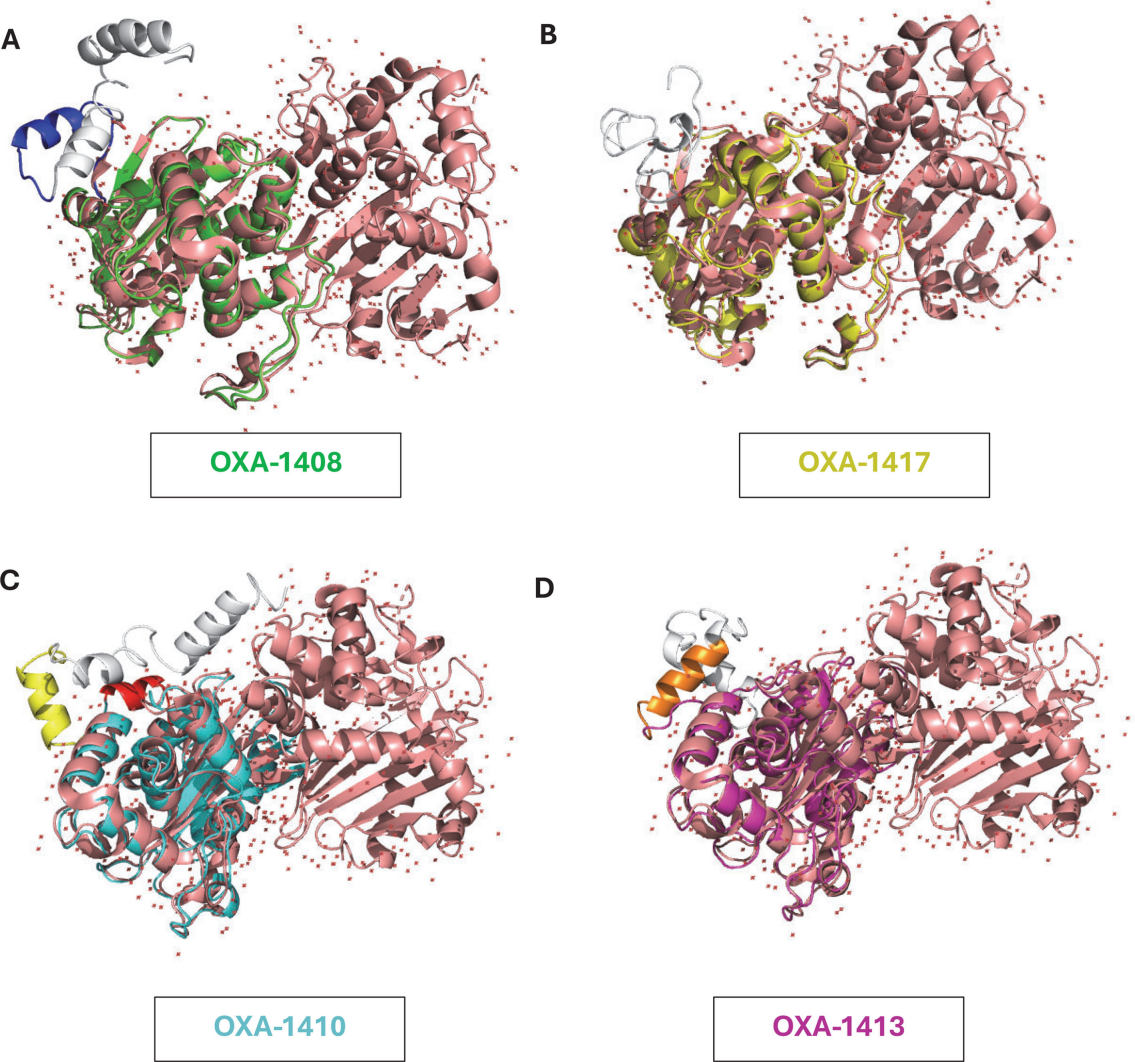
Protein structure prediction of OXA-48-like variants of *S. baltica* strains. Predicted tridimensional structures of OXA variants for (**A**) N1WShe5-IV (OXA-1408) and (**B**) T1WShe4 (OXA-1417), shown as examples of N-terminal deletions, and (**C**) N1WShe6 (OXA-1410) and (**D**) T1SShe5-III (OXA-1413), shown as examples of N-terminal insertions, and all aligned with the reference crystal structure of OXA-48 from *K. pneumoniae* (PDB: 3HBR), shown in light pink. The signal peptide in each structure is shown in gray, and alpha-helix variations in the N-terminal part of the protein are depicted in different colors.

To evaluate whether *bla*_OXA_ genes were under non-neutral selection in this *Shewanella* population, we carried out the Fu and Li test (D* = −0.56827, *P* > 0.10; F* = −0.85327, *P* > 0.10), which indicated non-significant deviation from neutrality. This was further corroborated by the Tajima test (D = −1.06065, *P* > 0.10), with this analysis evidencing codons with multiple evolutionary paths. An amino acid sequence alignment, using the sequence and structure of OXA-48 (PDB: 3HBR) and the OXA-551 sequence (CARD entry ARO: 3,005,775) as references, revealed diverse polymorphic sites across the *S. baltica* OXA proteins, preponderantly located in the N-terminal region of the mature enzyme (Fig. S1). This is exemplified in Fig. 1B with the OXA sequences of isolates N1WShe5-IV (OXA-1408), N1WShe6 (OXA-1410), T1SShe5-III (OXA-1413), and T1WShe4 (OXA-1417). To understand the structural impact of these mutations, we generated three-dimensional reconstructions of the representative divergent variants using deep-learning algorithms (26). Thus, compared with OXA-551, OXA-1408 and OXA-1417 exhibit deletions of five and eight amino acids, respectively (Fig. 1B), which affect the conformation of the first α-helix or results in its absence (Fig. 2A and B). In contrast, OXA-1410 and OXA-1413 carry insertions of five amino acids each (Fig. 1B), which, in the case of OXA-1410, leads to the formation of a second α-helix in the N-terminal region (Fig. 2C and D).

### N-terminal mutations and non-conservative substitutions are widespread in *Shewanella baltica* OXA-48-like enzymes

**Fig 3:**
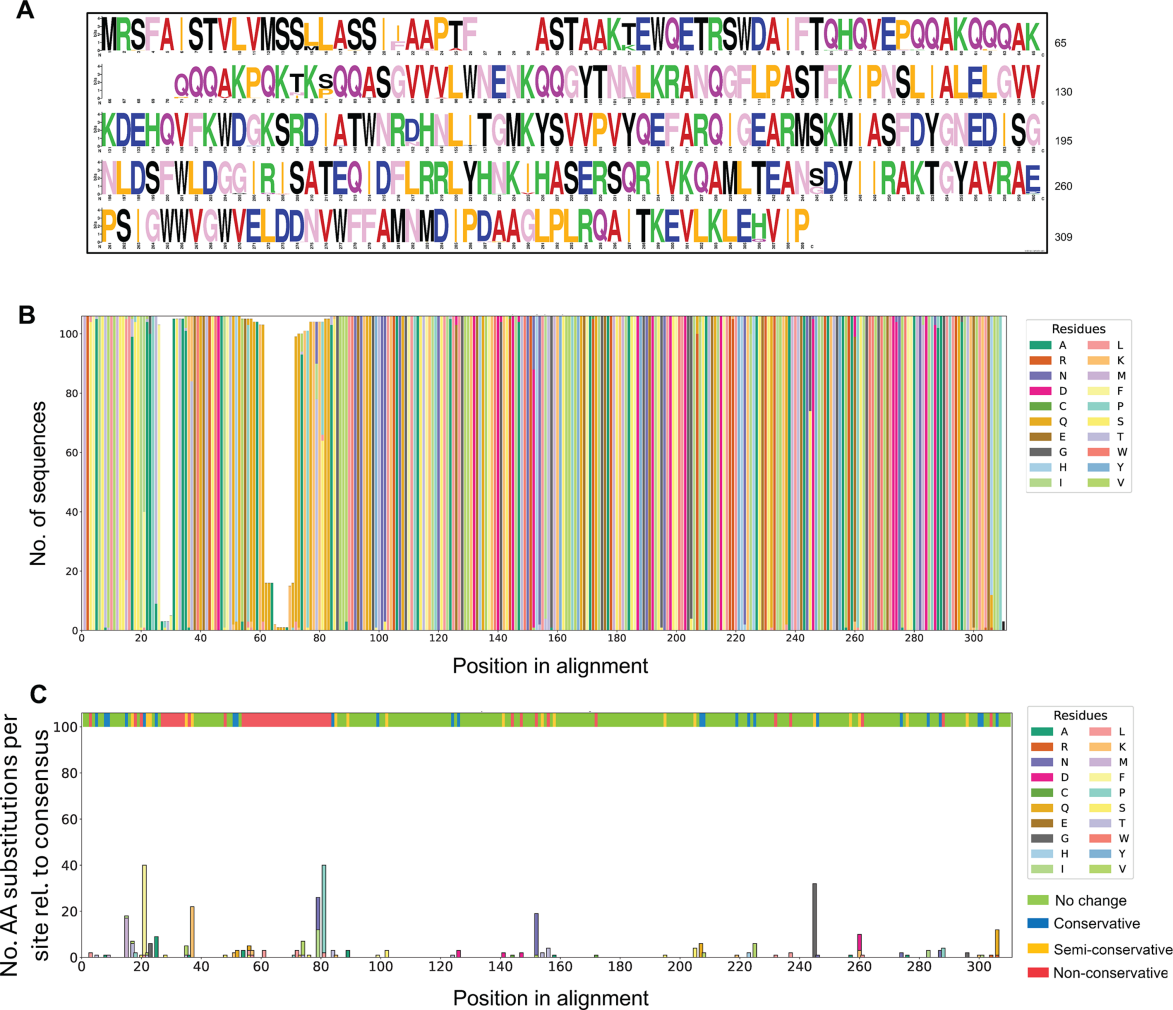
Variability of OXA-48-like sequences in *S. baltica* complex strains. (**A**) Multiple sequence alignment of the amino acid sequence obtained in this study, along with publicly available sequences from NCBI (81 genomes downloaded on 6 September 2025). Each sequence logo displays stacks of symbols representing the different amino acids, one for each position in the alignment, and the relative height of each letter indicates the frequency of the corresponding amino acid. (**B**) Stacked bar chart of amino acid residue frequencies per alignment position across the *N* = 106 sequences. Each bar shows the counts of residues observed at that site, color-coded by residue identity. (**C**) Distribution of amino acid substitutions relative to the consensus sequence (MRSFAISTVLVMSSLLASSIIAAPTFAPTFASTAAKTEWQETRSWDAIFTQHQVEPQQAKQQQAKQQQAKQQQAKPQKTKSQQASGVVVLWNENKQQGYTNNLKRANQGFLPASTFKIPNSLIALELGVVKDEHQVFKWDGKSRDIATWNRDHNLITGMKYSVVPVYQEFARQIGEARMSKMIASFDYGNEDISGNLDSFWLDGGIRISATEQIDFLRRLYHNKIHASERSQRIVKQAMLTEANSDYIIRAKTGYAVRAEPSIGWWVGWVELDDNVWFFAMNMDIPDAAGLPLRQAITKEVLKLEHVIP) across the 106 aligned sequences. At each alignment position, non-consensus residues are shown as stacked bars and are color-coded by residue ID. The horizontal bar above the plot represents the degree of conservation at each position, i.e., green indicates positions with no substitutions, blue denotes conservative substitutions, orange indicates semi-conservative substitutions, and red marks non-conservative substitutions.

To investigate whether the observed sequence variability was endemic to local *S. baltica* populations or broadly distributed in this species instead, we retrieved all *S. baltica* genomes from NCBI GenBank (*n* = 81, accessed 6 September 2025), extracted their chromosomal *bla*_OXA_ genes, and, together with the 25 sequences of this study, constructed a sequence logo based on aligned amino acid sequences (Fig. 3A). The frequency of amino acid residues at each position is shown in Fig. 3B. These reconstructions evidenced two regions that concentrated most amino acid substitutions, insertions, or deletions, located at positions 27–30 and 61–79 in the alignment. Key amino acids involved in catalysis by serine beta-lactamases such as OXA-48, namely the catalytic serine (position 114 in the sequence logo), carboxylated lysine (position 117), and the universal KTG motif (positions 252–254) (36) were highly conserved, presumptive of activity preservation despite sequence variation. Most amino acid deviations from the consensus sequence across the 106 analyzed OXA-48-like proteins (Fig. 3C) were non-conservative, consistent with the frequent insertions and deletions at the N-terminus.

For comparative purposes, we performed the same analysis on OXA sequences retrieved from well-represented *Shewanella* species in terms of genome sequence availability, with variable degrees of taxonomic relatedness with respect to *S. baltica*, namely *S. oncorhynchi* (*n* = 32; OXA-48-like), *S. xiamenensis* (*n* = 98; OXA-48-like), and *S. algae* (*n* = 219, OXA-55-like), which, in contrast to *S. baltica*, exhibited a much higher degree of homogeneity, reflected in fewer mutations and the absence of insertions or deletions (Fig. S2 to S4). This pattern could indicate higher intrinsic mutation rates in *S. baltica* compared with other *Shewanella* species, or alternatively, reflect the influence of selective pressures.

### Baltic Sea *Shewanella* exhibit discrete phenotypic resistance toward β-lactam antibiotics

**Fig 4:**
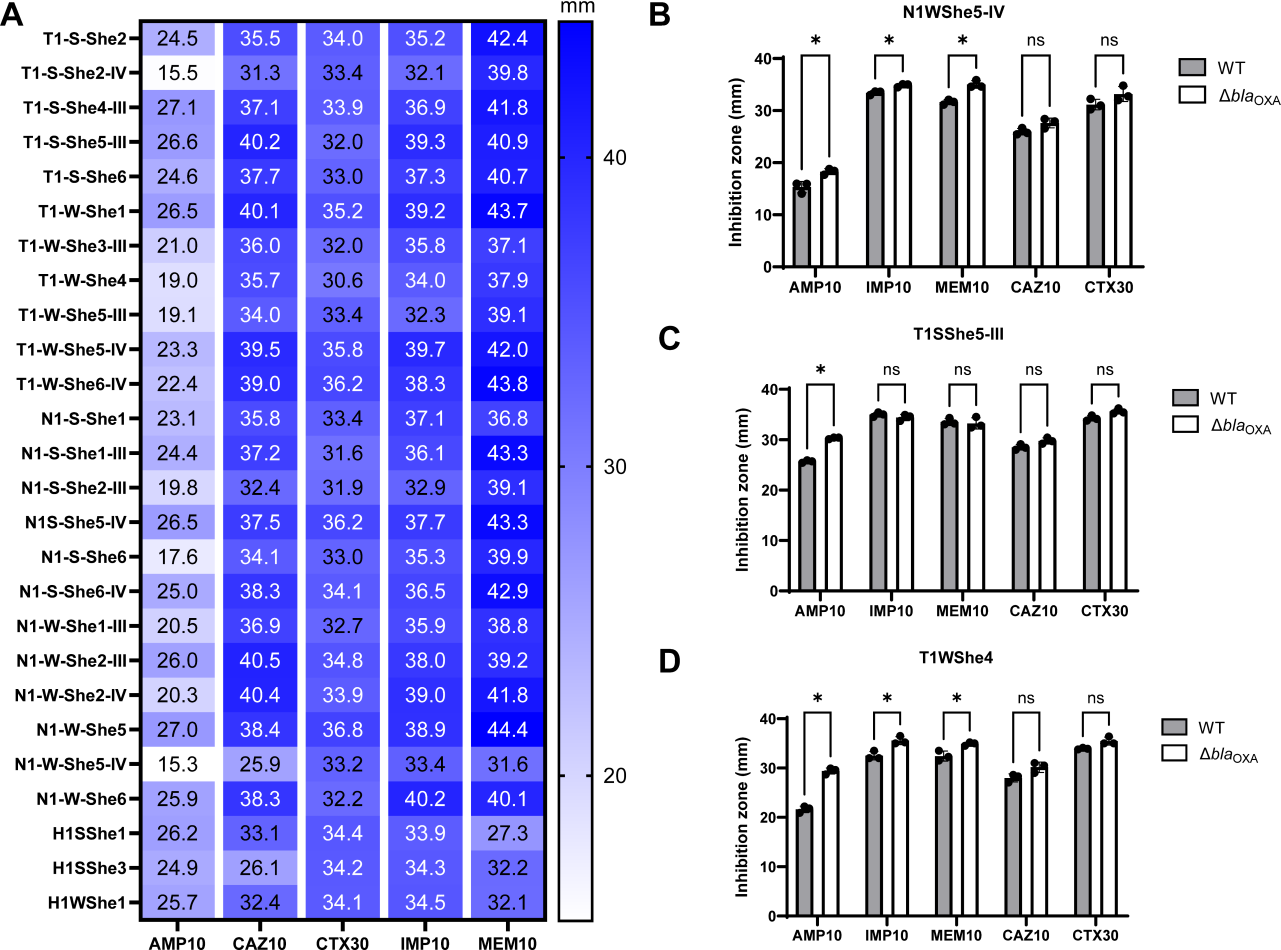
Susceptibility profiles of *S. baltica* complex isolates to β-lactam antibiotics and effect of *bla*_OXA_ deletion on antibiotic resistance. (**A**) Inhibition zones, determined by the disc diffusion method, of 25 *S. baltica* strains using discs loaded with ampicillin (AMP, 10 µg), ceftazidime (CAZ, 10 µg), cefotaxime (CTX, 30 µg), imipenem (IMP, 10 µg), and meropenem (MEM, 10 µg). (**B to D**) Comparative susceptibility data of N1WShe5-IV (OXA-1408), T1SShe5-III (OXA-1413), and T1WShe4 (OXA-1417) wild type (WT) and Δ*bla*_OXA_ mutants toward the same β-lactam antibiotics. The statistical significance of the differences was determined with unpaired *t*-tests, using *P* = 0.05 as threshold (*P < 0.05; ns = not significant).

Next, to investigate whether *bla*_OXA_ carriage was associated with phenotypic resistance in *S. baltica* isolates, the susceptibility profiles of the 25 strains were determined toward a panel of β-lactam antibiotics comprising ampicillin (AMP, 10 µg), meropenem (MEM, 10 µg), imipenem (IMP, 10 µg), ceftazidime (CAZ, 10 µg), and cefotaxime (CTX, 30 µg). The results of disc diffusion tests are presented in Fig. 4A. Growth inhibition diameters were in the range of 15.5–27.1 mm for AMP, 32.1–40.2 mm for MEM, 31.3–40.5 mm for IMP, 30.6–36.8 mm for CAZ, and 35.8–44.4 mm for CTX, which, in relative terms, given the absence of specific guidelines for the interpretation of disc diffusion tests with this group of microorganisms, evidenced a high degree of susceptibility to carbapenems (IMP, MEM) and third generation cephalosporins (CAZ, CTX), whereas susceptibility was comparatively lower toward the aminopenicillin ampicillin (AMP). Altogether, these results implied a rather discrete role of *bla*_OXA_ in *S. baltica* in mediating substantial resistance toward β-lactam antibiotics under our test conditions.

To further dissect the functional role of *bla*_OXA_ in *S. baltica*, we generated Δ*bla*_OXA_ in-frame deletion mutants from three representative strains, N1WShe5-IV (OXA-1408), T1SShe5-III (OXA-1413), and T1WShe4 (OXA-1417), which captures the structural diversity of the OXA variants within the studied strains (Fig. 1B; Fig. S1). Attempts to generate the same mutant in the N1WShe6 background failed because of the intrinsic resistance of this strain to sucrose-mediated counter-selection, which is widespread in *Shewanella*. Mutants were screened, in triplicate, against the same panel of antibiotics, using their respective wild-type (WT) strains as references. Our results revealed a statistically significant contribution of *bla*_OXA_ to AMP resistance in all strains (Fig. 4B through D), as well as to IMP and MEM in strains N1WShe5-IV and T1SShe5-III (Fig. 4C and D). Despite a seemingly modest biological impact, these findings confirm the expression and functional activity of the gene product under our experimental conditions.

### Heterologous expression of *Shewanella* OXA-551-like enzymes in *Escherichia coli* stimulates β-lactamase activity

**Fig 5:**
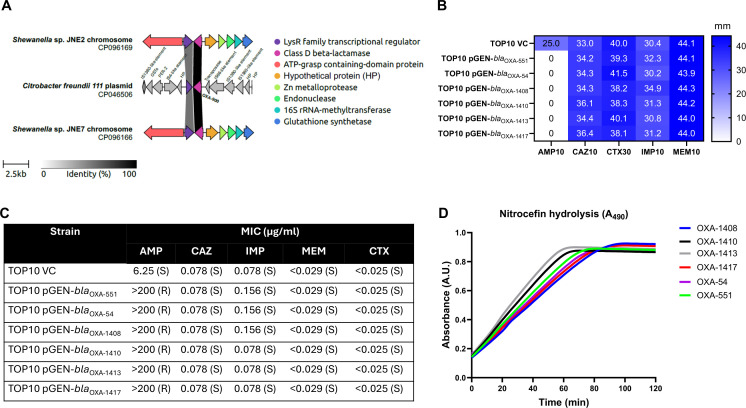
Performance of reference and novel OXA variants in a heterologous host. (**A**) Synteny plot showcasing the integration of *Shewanella* chromosomal *bla*_OXA_ and an adjacent LysR-type transcriptional regulation into an enterobacterial plasmid, illustrating horizontal transmission to *Enterobacteriaceae*. (**B**) Susceptibility of *Escherichia coli* TOP10 expressing OXA-551, OXA-54, OXA-1408, OXA-1410, OXA-1413, and OXA-1417, or carrying the empty cloning plasmid (VC, vector control) toward β-lactam antibiotics, expressed as inhibition zones from disc diffusion assays. (**C**) Minimal inhibitory concentrations of *E. coli* TOP10 strains toward β-lactam antibiotics, expressed as µg/mL (S = susceptible; R = resistant). (**D**) Kinetics of nitrocefin hydrolysis by whole-cell lysates of *E. coli* TOP10 strains expressing either OXA variant.

There is evidence that OXA β-lactamases originating from *Shewanella* spp. are currently widely distributed in enterobacterial multidrug resistance plasmids (Fig. 5A). To investigate the potential impact of the N-terminal mutations found in the OXA-551-like enzymes of our isolates on β-lactamase activity, we cloned the *bla*_OXA_ gene of the same four representative strains along with its native promoter sequences into a promoter-less plasmid. The same approach was taken for the cloning of OXA-551 and OXA-54, used as references. In both disk diffusion and broth microdilution assays, *E. coli* TOP10 expressing either OXA variant showed similar susceptibility to β-lactam antibiotics as the empty vector control (Fig. 5B and C). A clear exception was observed for ampicillin, where OXA expression enhanced the resistance phenotype, elevating MICs to >200 µg/mL.

The results from antibiotic susceptibility tests suggested a similar performance of the novel OXA variants with respect to OXA-551 and OXA-54. To further characterize this, we studied the kinetics of the hydrolytic degradation of nitrocefin, a chromogenic cephalosporin substrate, using cell lysates of *E. coli* TOP10 expressing either OXA-551, OXA-54, or the four exemplary variants OXA-1408, OXA-1410, OXA-1413, or OXA-1417. The results of this assay (Fig. 5D) demonstrated similar performance of all the enzymes, with the two variants carrying N-terminal insertions, OXA-1410 and OXA-1413, exhibiting somewhat higher activity than OXA-551, OXA-54, and their counterparts carrying N-terminal deletions. In addition, we tested CENTA (a chromogenic β-lactamase substrate) and imipenem (a carbapenem) and found that the variants analyzed in this study, including OXA-551 and OXA-54, showed negligible activity against these substrates under our experimental conditions (Fig. S5), which suggest a narrow substrate range for these enzymes.

**Fig 6:**
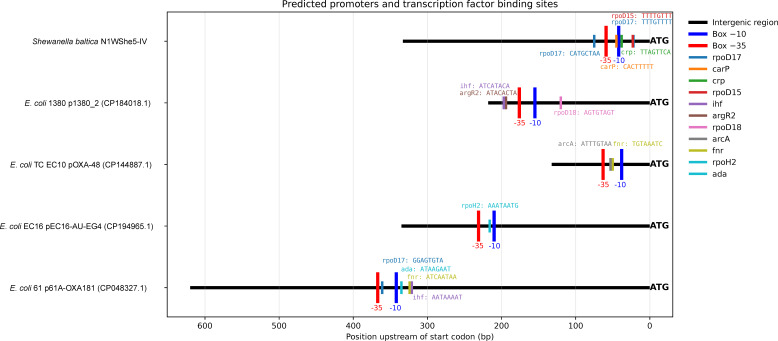
Promoter architecture of *bla*_OXA_ genes. Predictions of σ⁷⁰-type promoters and transcription factor binding sites for selected DNA regions upstream *bla*_OXA_ alleles are shown. Each horizontal black line represents the intergenic region upstream of a given *bla*_OXA_ open reading frame, oriented 5′−3′, with the start codon (ATG) indicated at the right end. Vertical lines mark key motifs: blue lines indicate −10 boxes, red lines indicate −35 boxes, and color-coded vertical ticks represent transcription factor binding sites. The scale is approximate.

As *bla*_OXA_ genes are not native to *E. coli*, we next interrogated factors regulating the expression of *in trans*-acquired *bla*_OXA-48_ alleles. Thus, we compared the promoter region of *S. baltica bla*_OXA_ with those of a set of *bla*_OXA_ alleles found in *E. coli* plasmids mediating antibiotic resistance (Fig. 6) BPROM analysis identified canonical σ^70^-type promoters upstream of all analyzed genes, although the predicted regulatory complexity varied markedly between sequences. While several promoters harbor binding sites for multiple global regulators, including, e.g., CRP, IHF, or RpoH, others show a more restricted regulatory architecture, with only binding sites for redox-associated regulators such as ArcA and FNR predicted in the promoter. Another signature of a putative distinct metabolic regulation of *bla*_OXA_ expression is the presence of predicted ArgR binding sites in certain promoters, suggesting regulation of gene expression by arginine metabolism. The *S. baltica* promoter displays a distinct organization, including different RpoD binding motifs and predicted binding sites for CRP and CarP (Fig. 6). The predicted binding of CarP putatively links *bla*_OXA_ expression with arginine and pyrimidine metabolism (37, 38). Noteworthily, two of the exemplary promoters from *E. coli* plasmids (found in sequences CP194965.1 and CP048327.1, Fig. 6) are predicted to have unusually long 5′ untranslated regions (5′ UTRs), which might affect mRNA stability and OXA expression.

## DISCUSSION

AMR represents one of the greatest public health concerns of our times, with an estimate of over 10 million annual deaths by the year 2050 directly attributable to or associated with infections caused by drug-resistant pathogens (39). With broad spectrum penicillins and cephalosporins being the most prescribed antibiotics worldwide (40), the dissemination of β-lactamase resistance across bacterial pathogens is a growing concern. OXA-48-like β-lactamases are particularly problematic because of the low levels of phenotypic resistance associated with their carriage *in vitro*, despite evidence linking *bla*_OXA_ carriage with failure of carbapenem-based antimicrobial chemotherapy (41). Our study showed, indeed, that the phenotypic resistance of *S. baltica*-like strains naturally carrying a chromosomally encoded *bla*_OXA_ gene (which is a widespread genomic characteristic in the genus *Shewanella* [3]) was weak toward a panel of diverse β-lactam antibiotics including ampicillin, which is typically proficiently hydrolyzed by OXA-48-like enzymes (42, 43). This is not uncommon and could be related to variability in OXA expression under the experimental conditions. For example, a recent study involving diverse carbapenem-susceptible and a carbapenem-resistant *S. algae* isolates demonstrated higher transcript levels of *bla*_OXA_ mRNA in the resistant isolate as compared with the susceptible strains, despite all strains carrying minimally different *bla*_OXA_ alleles (4). This is also consistent with our mutational analyses, with Δ*bla*_OXA_ deletion mutants displaying, overall, relatively discrete susceptibility defects toward β-lactam antibiotics as compared with the corresponding WT parental strains.

While there is wide agreement on *Shewanella* spp. as progenitors of OXA-48-like β-lactamases, it is less clear whether these enzymes have mobilized from the chromosome of the *Shewanella* donors into enterobacterial plasmids directly or through intermediate hosts (44). OXA-48-like enzymes preponderantly disseminate across clinically relevant *Enterobacterales* within broad-host conjugative IncL/M-type conjugative plasmids associated with Tn*1999* insertion (9, 45). Other OXA-48-like variants originating from *Shewanella* have been found in plasmids with other replicon types, including ColE2, IncX2, IncN1, IncT and IncA/C (9). Novel allelic variants of *Shewanella*-derived OXA-48-like enzymes are emerging, in part due to increasing surveillance and sequencing efforts, raising clinical concerns (46). A heterologous analysis of *Shewanella oneidensis* OXA-54 has previously been performed through *in trans* expression under a *lac* promoter on a high-copy-number plasmid (18). In our study, we employed a nature-mimicking approach to simulate the horizontal acquisition of *S. baltica bla*_OXA_ by an enterobacterial host. To that end, instead of expressing the gene from an inducible promoter, we cloned native genes and their promoter regions into a low copy-number, promoter-less plasmid that is stable in *E. coli* in the absence of antibiotic selective pressure (47). This approach circumvents the bias introduced by protein overexpression from *E. coli*-adapted promoters or high copy number expression shuttles, allowing a more realistic assessment of phenotypic resistance acquisition. We showed that, compared with the phenotypic resistance exhibited by the native hosts, expression of *S. baltica* OXA-48-like in *E. coli* stimulates phenotypic resistance against AMP while displaying discrete phenotypic resistance to carbapenems or third generation cephalosporins, consistent with phenotypic evidence of OXA-48-like carriage by clinical strain subsets (48) or heterologous functional studies with other *Shewanella* OXA-48-like enzymes (46).

OXA enzymes show remarkable sequence diversity. In *Shewanella*, the phylogenetic distribution of OXA-48-like carbapenemases resembles the taxonomy of the genus, suggesting parallel evolution across different taxa (2). We have shown here that *S. baltica* and closely related species harbor an array of OXA-48-like variants that differ primarily in their N-termini, in some cases presenting in-frame insertions or deletions. In fact, of the 25 strains of this study, 20 carried a novel OXA variant, which showcases the still unknown environmental diversity pool of ARGs and the evolutionary forces that drive their diversification. Through a comparative analysis, we have shown that *S. baltica* OXA-48-like enzymes are substantially more variable and accumulate more non-conservative mutations than those encoded in the genomes of other *Shewanella* species. From a genomic standpoint, *S. baltica* is known to be a highly heterogeneous and recombination-prone clade (21, 49–51), in contrast to *S. algae*, which is substantially more uniform (50, 51). The higher degree of sequence variability in *S. baltica* OXA-48-like enzymes could indicate higher intrinsic mutation frequency in this species, which is currently unknown, although it could also be the result of evolutionary forces favoring the propagation of N-terminal mutations.

In OXA-48-like enzymes, residues in or around the β5–β6 loop and Ω loop are important determinants of enzymatic activity and substrate specificity (52–55). While none of these regions are in the N-terminus of the protein, the high degree of N-terminal sequence and structural divergence has been previously highlighted in novel OXA-48 variants (56). Given the *prima facie* marginal contribution to phenotypic resistance in the native host, we reasoned that mutations affecting enzyme function might have minimal fitness consequences. We therefore studied whether rearrangements in the N-terminal region of *S. baltica* OXA proteins could potentially affect enzyme performance. Computational analyses using four representative OXA enzymes carrying five amino acid insertions or deletions with respect to the closest OXA-48-like subtype, OXA-551, predicted structural alterations affecting N-terminal α-helices. Our experimental analyses of OXA-1408, OXA-1410, OXA-1413, and OXA-1417 demonstrated that in-frame alterations in this region do not substantially affect β-lactamase activity *in vivo* when expressed in a heterologous host. Similarly, *in vitro* antibiotic hydrolysis assays showed that the enzymes harboring insertions exhibit a modestly increased hydrolytic capacity than OXA-551, OXA-54, or enzymes carrying N-terminal deletions. The lack of non-deleterious mutations also implies that, despite their overall low contribution to phenotypic resistance, OXA enzymes may play a significant role in the eco-physiology of *Shewanella* and support the propagation of mutant variants without functional consequences across natural populations.

Promoter architecture and regulatory variability have emerged as important determinants of ARG expression (57). Promoter sequence variants of clinically relevant ARGs, including *bla_OXA-48_*, are associated with differences in expression levels under various environmental conditions, and specific transcription factors, such as FNR, ArcA or ArgR, can influence expression in response to distinct metabolic or stress signals (57). Experimental work in other systems has also demonstrated that point mutations within core promoter elements, such as the −10 box, can modulate β-lactamase expression and resistance levels, as seen for the *bla*_OXA-61_ promoter in *Campylobacter jejuni* (58) and in chromosomal β-lactamase promoters of *Klebsiella oxytoca* where alterations in −10 and −35 motifs significantly changed promoter strength (59). Indeed, the clinical impact of *bla*_OXA_ genes is largely influenced by the strength of the promoter driving their transcription (60). The acquisition of certain insertion sequences may provide highly optimized −35 and −10 hexamers that constitutively upregulate mRNA levels (61–63). Consequently, the presence of a *bla*_OXA_ gene alone is often insufficient to predict a resistant phenotype; rather, it is the genetic context and the recruitment of these elements that ultimately determine the level of enzyme production and the resulting MICs. Interestingly, high expression levels of OXA-48 carbapenemases have been found to impose a fitness costs associated with resistance plasmid acquisition by *Enterobacterales*, with intense transcriptional activity leading to collateral sensitivity to other antibiotics like colistin and azithromycin (64, 65).

Horizontal mobilization of ARGs can include adjacent upstream sequences, such as parts of the native promoter region (55). In these cases, binding sites for transcription factors present in the original chromosomal context would also be co-transferred, making them potentially available to influence gene expression in the new host. In this study, we have shown that the *S. baltica bla*_OXA_ promoter is predicted to accommodate the binding of global regulators, such as RpoD and CRP, as well as CarP. In *E. coli*, CarP acts as a pyrimidine-mediated repressor of *carAB* transcription (37, 38), which encodes carbamoyl phosphate synthase, a key enzyme in arginine and pyrimidine biosynthesis, thereby potentially extending previously proposed links between *bla*_OXA_ expression and arginine metabolism (57). Of note, CarP is also necessary for the resolution of ColE1 plasmid multimers (37). Besides, in our comparative analysis of the *S. baltica bla*_OXA_ promoter with respect to *E. coli* plasmid-borne counterparts, we identified some unusually long 5′ UTRs (>200–300 nt), as predicted by BPROM. Such lengths are atypical, considering that the length of most 5′ UTRs in *E. coli* genes is 25–35 nt, although 5′ UTRs as long as 700 bp have also been reported (66). The length of 5′ UTRs have profound effects on mRNA stability and translation (67–69), which could potentially relate to the known variability in the levels of phenotypic resistance associated with *bla*_OXA_ carriage in different bacterial hosts and cellular physiological contexts (12). While *in silico* predictions require empirical validation, these initial observations warrant further investigation.

Taken together, this work reaffirms *Shewanella* communities as a substantial reservoir of OXA diversity in aquatic environments, prone to potential horizontal dissemination. By genomically and functionally characterizing the novel variants presented here, our findings emphasize the need for continued environmental surveillance to monitor the transfer of these resistance determinants into clinical pathogens.

## Data Availability

*Shewanella* genome sequences are available in NCBI GenBank under accessions PX283710 to PX283731 and PX713573 to PX713575.

## SUPPLEMENTAL MATERIAL

File S1 aem.00125-26-s0001.xlsx GenBank accession numbers for the *Shewanella* genomes used in generating Fig. 3 and Fig. S2 to S4.

Supplemental material aem.00125-26-s0002.pdf Fig. S1 to S5; Tables S1 and S2.

ASM does not own the copyrights to Supplemental Material that may be linked to, or accessed through, an article. The authors have granted ASM a non-exclusive, world-wide license to publish the Supplemental Material files. Please contact the corresponding author directly for reuse.
