# Supplementary material for: Diversity and function of OXA-48-like β-lactamase variants in environmental Shewanella isolates from Stockholm, Sweden
Source: Appl Environ Microbiol. 2026 May 21;92(6):e00125-26. doi: 10.1128/aem.00125-26 (PMC13274421; doi:10.1128/aem.00125-26)
Supplement: Supplemental material — Fig. S1 to S5; Tables S1 and S2. [file aem.00125-26-s0002.pdf]

Diversity and function of OXA-48-like β-lactamase variants in environmental *Shewanella* isolates from Stockholm, Sweden

Víctor Fernández-Juárez[1], Marija Petrovic[1], I. Mihindukulasooriya[1], Enrique Joffré[1,2], Åsa Sjöling[1,3], and Alberto J. Martín-Rodríguez[1,4*]

[1] Department of Microbiology, Tumor and Cell Biology, Karolinska Institutet, Stockholm, Sweden

[2] Department of Medical Biochemistry and Microbiology, Uppsala University, Uppsala, Sweden

[3] Department of Chemistry and Molecular Biology, University of Gothenburg, Gothenburg, Sweden

[4] Department of Clinical Sciences, University of Las Palmas de Gran Canaria, Las Palmas de Gran Canaria, Spain

Correspondence: jonatan.martin.rodriguez@ki.se or alberto.martin@ulpgc.es

SUPPLEMENTAL INFORMATION

Page 2: Figure S1. Sequence alignment of the 25 full-length OXA proteins of the *Shewanella* strains of this study.

Page 4: Figure S2. Variability of OXA enzymes in *S. algae*.

Page 5: Figure S3. Variability of OXA enzymes in *S. oncorhynchi*.

Page 6: Figure S4. Variability of OXA enzymes in *S. xiamenensis*.

Page 7: Figure S5. Hydrolysis assays with CENTA and imipenem.

Page 8: Table S1. Strains used in this study.

Page 11: Table S2. Plasmids and primers used in this study.

Page 12: Supplemental references.

Figure S1. Sequence alignment of the 25 full-length OXA proteins of the *Shewanella* strains of this study. The crystal structure of OXA-48 (PDB entry 3HBR) was used as a reference for secondary structure elements. The OXA-551 sequence (CARD entry ARO:3005775), most closely related to *S. baltica* OXA enzymes, is included as a reference.

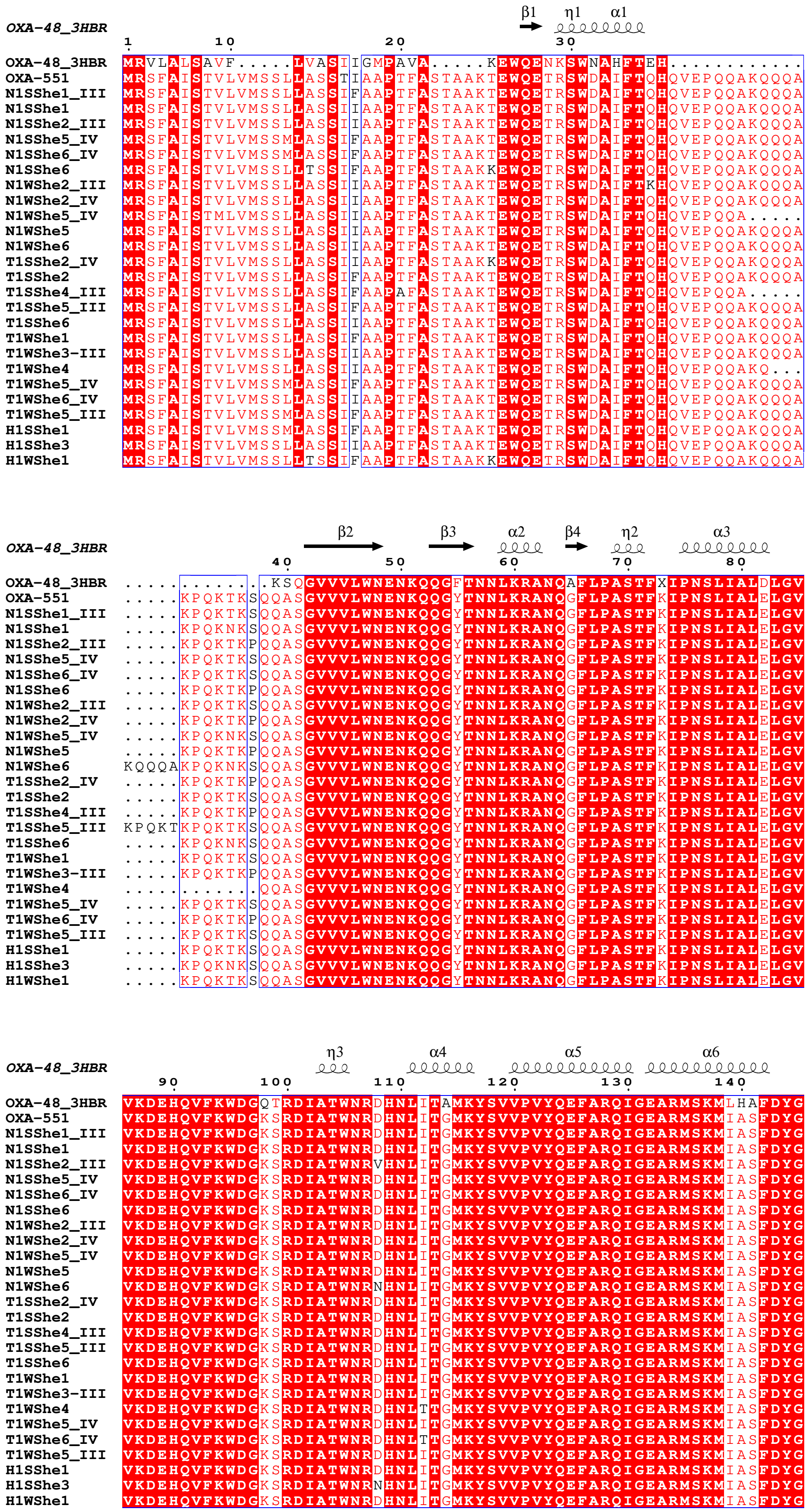

Figure S1 (cont).

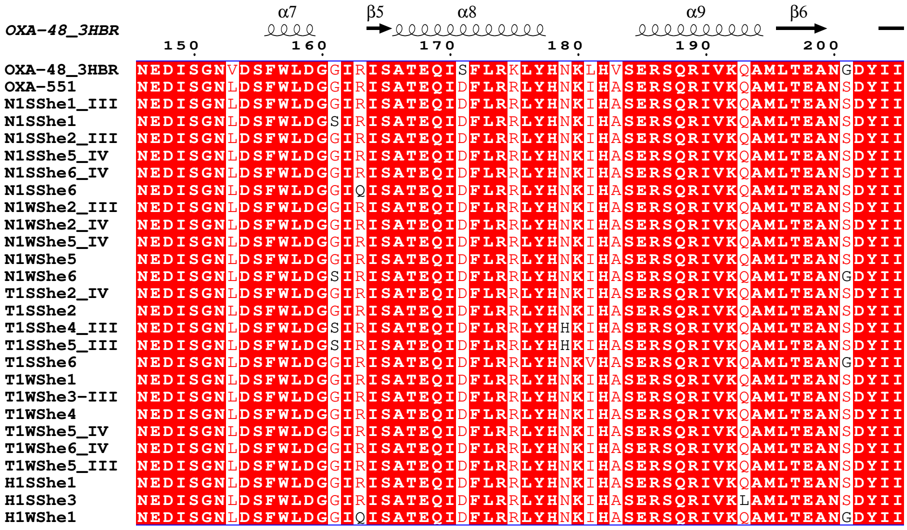

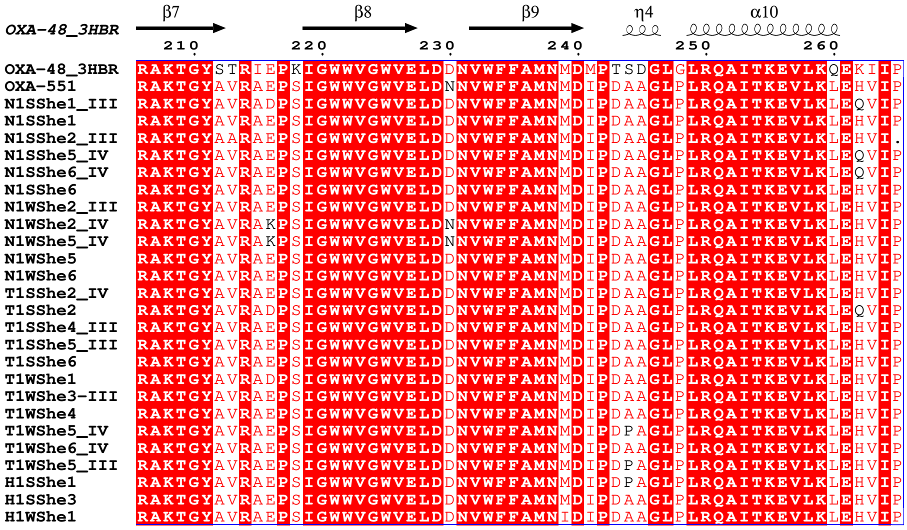

Figure S2. Variability of OXA enzymes in *S. algae*. A. Multiple sequence alignment of the amino acid sequence obtained in this study, along with publicly available sequences from NCBI (219 genomes, downloaded on May 31, 2025). Each sequence logo displays stacks of symbols representing the different amino acids, one for each position in the alignment, and the relative height of each letter indicates the frequency of the corresponding amino acid. B. Stacked bar chart of amino acid residue frequencies per alignment position across the N = 219 sequences. Each bar shows the counts of residues observed at that site, color-coded by residue identity. **C.** Distribution of amino acid substitutions relative to the consensus sequence (MNKGLHRKRLSKRLLLPMLLCLLAQQTQAVAAEQTKVSDVGSEVTAEG WQEVRRWDKLFESAGVKGSLLLWDQKRSLGLSNNLSRAAEGFIPASTFKIPSSLIALETGAV RDETSRFSWDGKVREIAAWNRDQSFRTAMKYSVVPVYQQLAREIGPKVMAAMVRQLDYGN QDIGGQADSFWLDGQLRITAFQQVDFLRQLHDNKLPVSERSQRIVKQMMLTEASTDYIIRAK TGYGVRRTPAIGWWVGWLELDDNTVYFAVNLDLASASQLPLRQQLVKQVLKQEQLLP) across the 219 aligned sequences. At each alignment position, non-consensus residues are shown as stacked bars and are color-coded by residue ID. The horizontal bar above the plot represents the degree of conservation at each position, i.e., green indicates positions with no substitutions, blue denotes conservative substitutions, orange indicates semi-conservative substitutions, and red marks non-conservative substitutions.

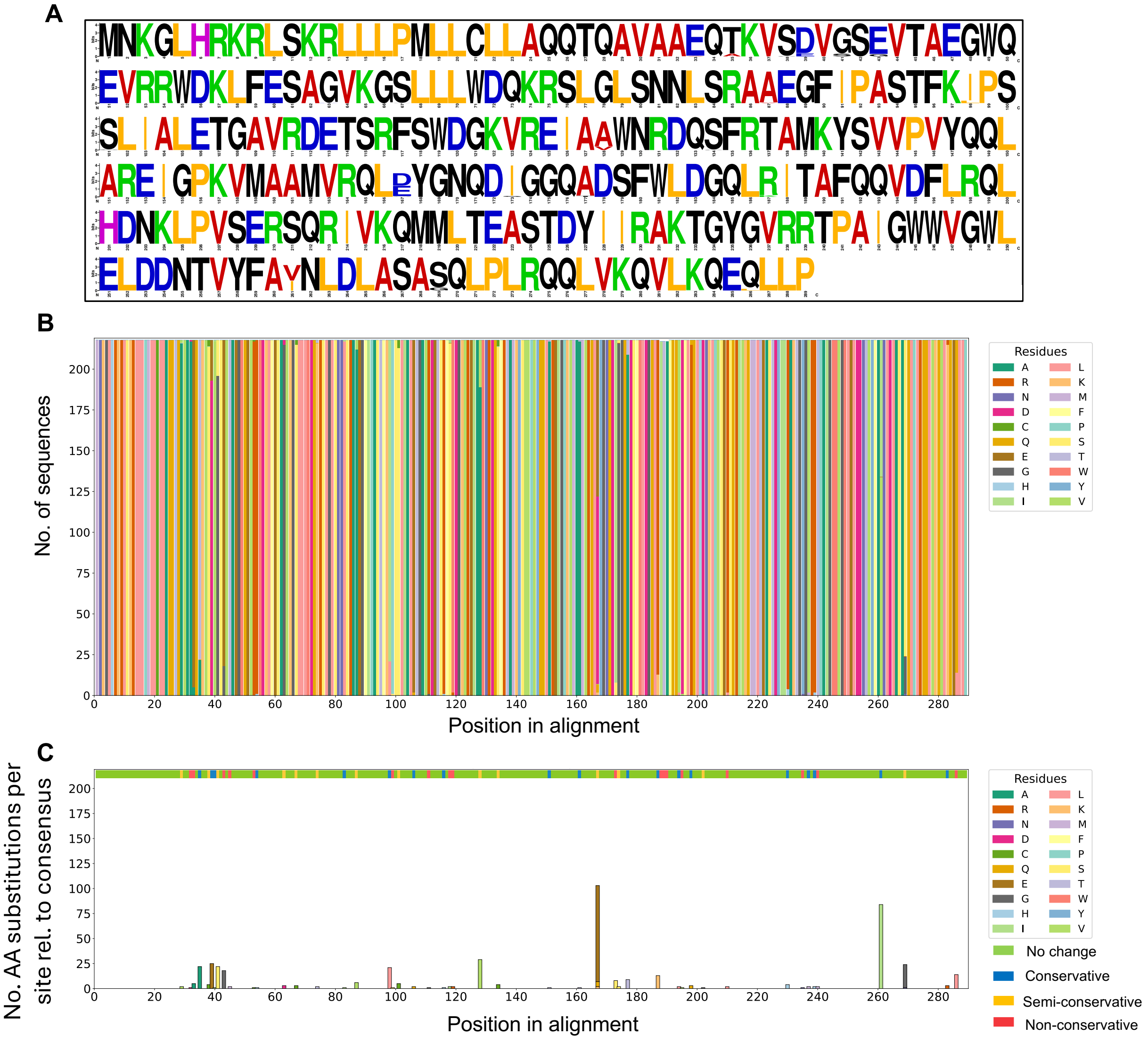

Figure S3. Variability of OXA enzymes in *S. oncorhynchi.* A. Multiple sequence alignment of the amino acid sequence obtained in this study, along with publicly available sequences from NCBI (33 genomes, downloaded on May 31, 2025). Each sequence logo displays stacks of symbols representing the different amino acids, one for each position in the alignment, and the relative height of each letter indicates the frequency of the corresponding amino acid. B. Stacked bar chart of amino acid residue frequencies per alignment position across the N = 33 sequences. Each bar shows the counts of residues observed at that site, color-coded by residue identity. **C.** Distribution of amino acid substitutions relative to the consensus sequence (MRLFTISAVLVMSSILVSSSLVAPTFASTTAKEWQETRSWDASFTQHQ TKGVVVLWNENKQQGFTNNLKRANQGFLPASTFKIPNSLIALELGVVKDEHQVFKWDGKSR DIATWNRDHNLITAMKYSVVPVYQEFARQIGEARMSKMIASFDYGNEDISGNLDSFWLDGGI RISATEQIDFLRKLYHNKIHASERSLRIVKQAMLTEANSDYIIRAKTGYAVRAEPSIGWWVGWV ELDDNVWFFAMNMDIPDAAGLPLRQAITKEILKRERVIP*) across the 33 aligned sequences. At each alignment position, non-consensus residues are shown as stacked bars and are color-coded by residue ID. The horizontal bar above the plot represents the degree of conservation at each position, i.e., green indicates positions with no substitutions, blue denotes conservative substitutions, orange indicates semi-conservative substitutions, and red marks non-conservative substitutions.

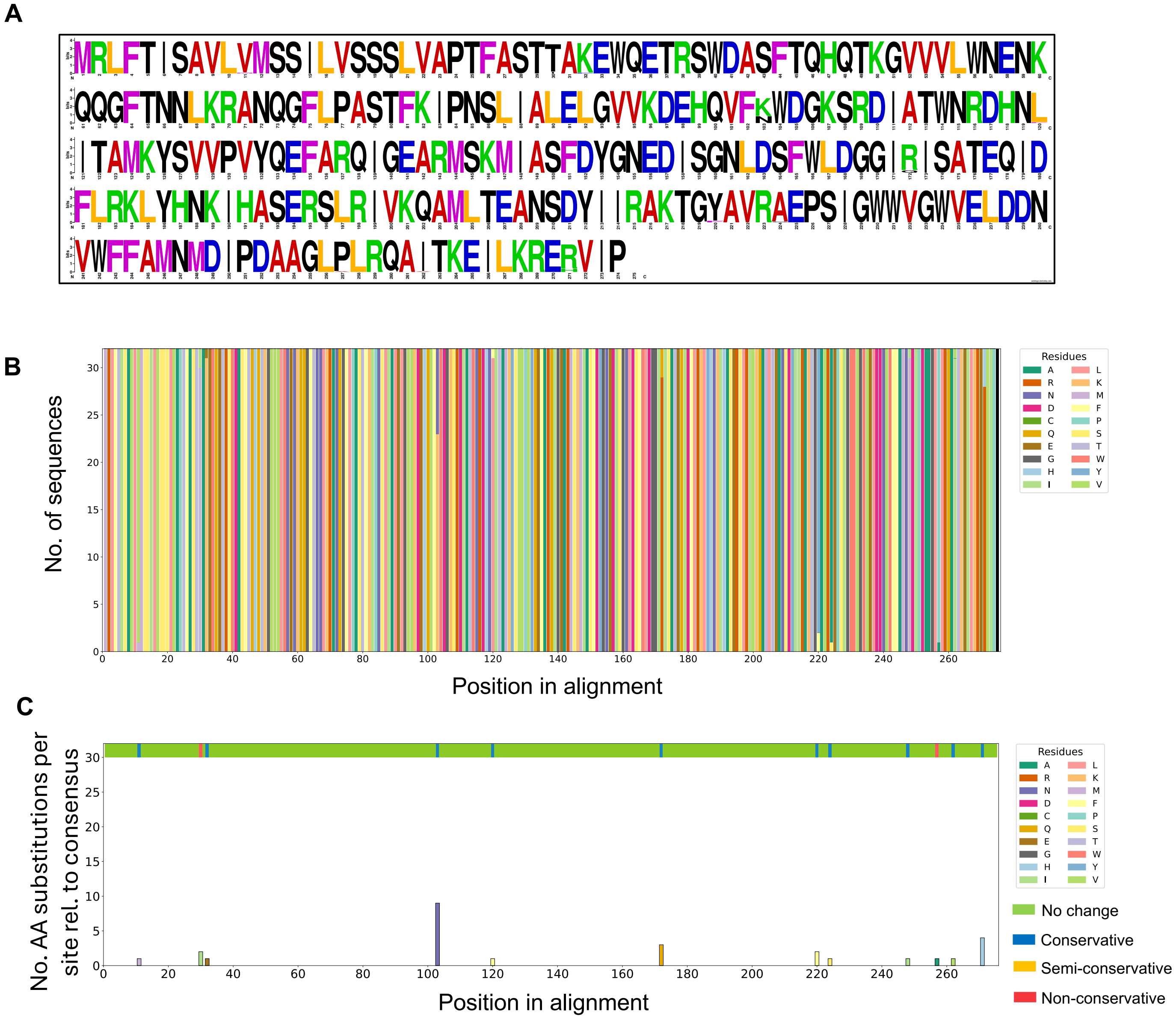

Figure S4. Variability of OXA enzymes in *S. xiamenensis.* A. Multiple sequence alignment of the amino acid sequence obtained in this study, along with publicly available sequences from NCBI (98 genomes, downloaded on May 31, 2025). Each sequence logo displays stacks of symbols representing the different amino acids, one for each position in the alignment, and the relative height of each letter indicates the frequency of the corresponding amino acid. B. Stacked bar chart of amino acid residue frequencies per alignment position across the N = 98 sequences. Each bar shows the counts of residues observed at that site, color-coded by residue identity. **C.** Distribution of amino acid substitutions relative to the consensus sequence (MRVLALSAVFLVASIIGMPAVAKEWQENKSWNAHFTEHKSQGV VVLWNENKQQGFTNNLKRANQAFLPASTFKIPNSLIALDLGVVKDEHQVFKWDGQTRDIAT WNRDHNLITAMKYSVVPVYQEFARQIGEARMSKMLHAFDYGNEDISGNVDSFWLDGGIRIS ATEQISFLRKLYHNKLHVSERSQRIVKQAMLTEANGDYIIRAKTGYSTRIEPKIGWWVGWVEL DDNVWFFAMNMDMPTSDGLGLRQAITKEVLKQEKIIP*) across the 98 aligned sequences. At each alignment position, non-consensus residues are shown as stacked bars and are color-coded by residue ID. The horizontal bar above the plot represents the degree of conservation at each position, i.e., green indicates positions with no substitutions, blue denotes conservative substitutions, orange indicates semi-conservative substitutions, and red marks non-conservative substitutions.

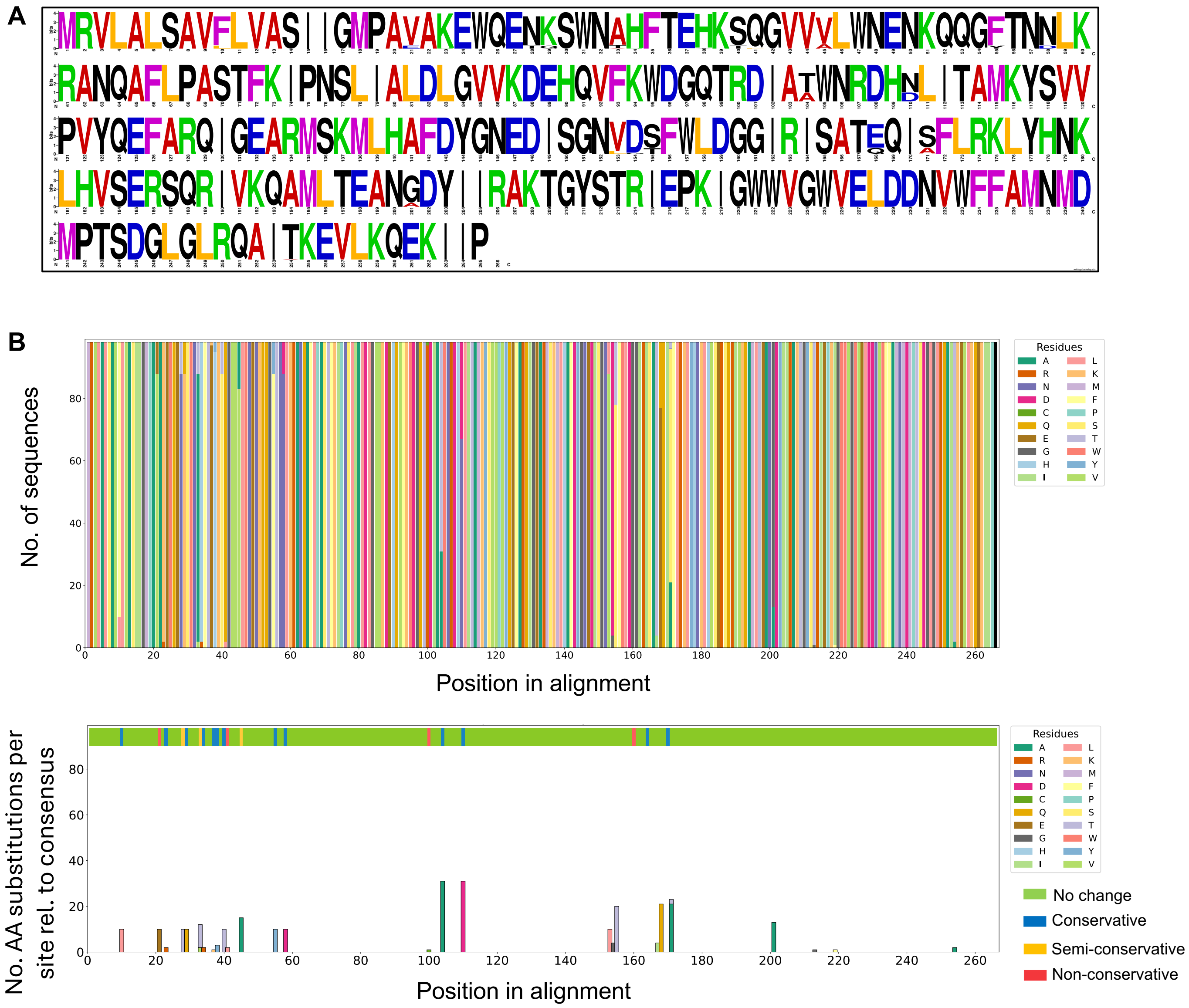

Figure S5. Hydrolysis tests for CENTA and imipenem. Shown are exemplary hydrolysis reactions by OXA-551, OXA-54, and OXA-1408 for the chromogenic cephalosporin CENTA (top row), evidenced by a change from pale yellow to dark yellow, and for imipenem (bottom row), evidenced by the Blue-Carba method as a change from dark blue to green. The empty vector control (VC) and a blank were included in both tests as negative controls, alongside the ESBL CTX-M-55, which is known to hydrolyze both substrates and thus served as a positive control.

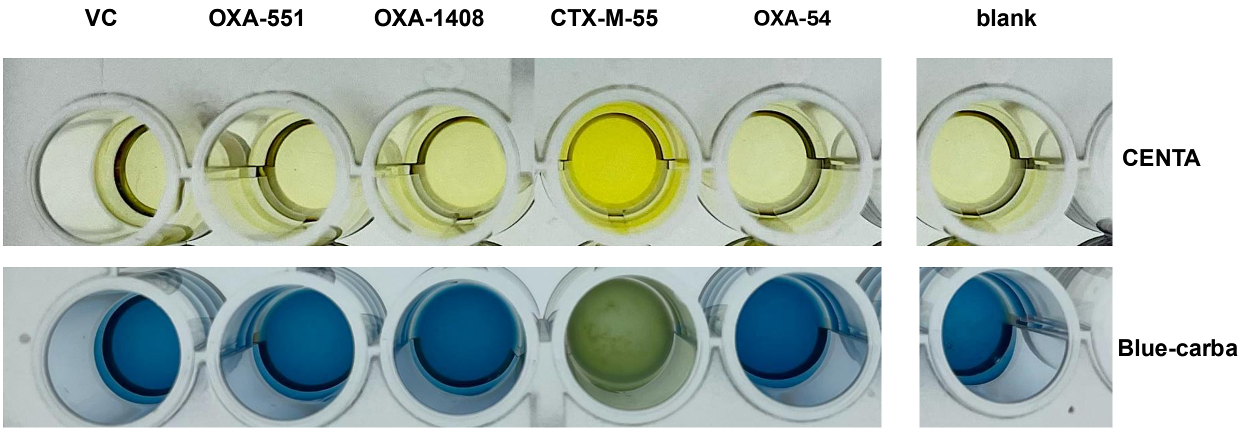

Table S1. Strains used in this study.

Strain	Description	OXA enzyme	Source or reference
Escherichia coli MFDpir	Diaminopimelic acid auxotrophic strain used in bi-parental mating	-	(1)
Escherichia coli TOP10	General-purpose cloning strain	-	Invitrogen
Shewanella sp. N1SShe1	*Shewanella* sp. isolated from Baltic Sea sediments (Tyresö, Sweden)	OXA-1402	This study
Shewanella sp. N1SShe1-III	*Shewanella* sp. isolated from Baltic Sea sediments (Tyresö, Sweden) on agar containing colistin (8 µg/ml)	OXA-1401	This study
Shewanella sp. N1SShe2-III	*Shewanella* sp. isolated from Baltic Sea sediments (Tyresö, Sweden) on agar containing colistin (8 µg/ml)	OXA-1403	This study
Shewanella sp. N1SShe5-IV	*Shewanella* sp. isolated from Baltic Sea sediments (Nynäshamn, Sweden) on agar containing meropenem (16 µg/ml)	OXA-1404	This study
Shewanella sp. N1SShe6	*Shewanella* sp. isolated from Baltic Sea sediments (Nynäshamn, Sweden)	OXA-1405	This study
Shewanella sp. N1SShe6-IV	*Shewanella* sp. isolated from Baltic Sea sediments (Nynäshamn, Sweden) on agar containing meropenem (16 µg/ml)	OXA-1404	This study
Shewanella sp. N1WShe2-III	*Shewanella* sp. isolated from Baltic Sea water (Nynäshamn, Sweden) on agar containing colistin (8 µg/ml)	OXA-1406	This study
Shewanella sp. N1WShe2-IV	*Shewanella* sp. isolated from Baltic Sea water (Nynäshamn, Sweden) on agar containing meropenem (16 µg/ml)	OXA-1407	This study
Shewanella sp. N1WShe5	*Shewanella* sp. isolated from Baltic	OXA-1409	This study

	Sea water (Nynäshamn, Sweden)		
Shewanella sp. N1WShe5-IV	*Shewanella* sp. isolated from Baltic Sea water (Nynäshamn, Sweden) on agar containing meropenem (16 µg/ml)	OXA-1408	This study
Shewanella sp. N1WShe5-IV Δbla_{OXA}	Mutant derivative of *Shewanella* sp. N1WShe5-IV	-	This study
Shewanella sp. N1WShe6	*Shewanella* sp. isolated from Baltic Sea water (Nynäshamn, Sweden)	OXA-1410	This study
Shewanella sp. T1SShe2	*Shewanella* sp. isolated from Baltic Sea sediments (Tyresö, Sweden)	OXA-1401	This study
Shewanella sp. T1SShe2-IV	*Shewanella* sp. isolated from Baltic Sea sediments (Tyresö, Sweden) on agar containing meropenem (16 µg/ml)	OXA-1411	This study
Shewanella sp. T1SShe4-III	*Shewanella* sp. isolated from Baltic Sea sediments (Tyresö, Sweden) on agar containing colistin (8 µg/ml)	OXA-1412	This study
Shewanella sp. T1SShe5-III	*Shewanella* sp. isolated from Baltic Sea sediments (Tyresö, Sweden) on agar containing colistin (8 µg/ml)	OXA-1413	This study
Shewanella sp. T1SShe5-III Δbla_{OXA}	Mutant derivative of *Shewanella* sp. T1SShe5-III	-	This study
Shewanella sp. T1SShe6	*Shewanella* sp. isolated from Baltic Sea sediments (Tyresö, Sweden)	OXA-1414	This study
Shewanella sp. T1WShe1	*Shewanella* sp. isolated from Baltic Sea water (Tyresö, Sweden)	OXA-1416	This study
Shewanella sp. T1WShe3-III	*Shewanella* sp. isolated from Baltic Sea water (Tyresö, Sweden) on agar containing colistin (8 µg/ml)	OXA-1409	This study

Shewanella sp. T1WShe4	*Shewanella* sp. isolated from Baltic Sea water (Tyresö, Sweden)	OXA-1417	This study
Shewanella sp. T1WShe4 Δbla_{OXA}	Mutant derivative of *Shewanella* sp. T1WShe4	-	This study
Shewanella sp. T1WShe5-III	*Shewanella* sp. isolated from Baltic Sea water (Tyresö, Sweden) on agar containing colistin (8 µg/ml)	OXA-1415	This study
Shewanella sp. T1WShe5-IV	*Shewanella* sp. isolated from Baltic Sea water (Tyresö, Sweden) on agar containing meropenem (16 µg/ml)	OXA-1415	This study
Shewanella sp. T1WShe6-IV	*Shewanella* sp. isolated from Baltic Sea water (Tyresö, Sweden) on agar containing meropenem (16 µg/ml)	OXA-1418	This study
Shewanella H1SShe1	*Shewanella* sp. isolated from Lake Brunnsviken shoreline sediments	OXA-1415	This study
Shewanella H1SShe3	*Shewanella* sp. isolated from Lake Brunnsviken shoreline sediments	OXA-1428	This study
Shewanella H1WShe1	*Shewanella* sp. isolated from Lake Brunnsviken water	OXA-1429	This study

Table S2. Plasmids and primers used in this study.

Plasmid or primer	Description or sequence	Source or reference
**Plasmids**		
pKNG101	Suicide vector for allelic exchange, R6Kori, Sm^R, *sacB*	(2)
pGEN-MCS-Tc	pGEN-MCS derivative in which the *bla* cassette has been replaced with a *tet* cassette and its promoter (XbaI/SpeI sites)	This study. Derivative of Addgene plasmid #44919 (3)
pGEN-*bla*$_{OXA\text{-}551}$	Expression plasmid for the reference OXA-551 enzyme under its native *Shewanella* promoter	This study
pGEN-*bla*$_{OXA\text{-}54}$	Expression plasmid for the novel OXA-54 enzyme under its native *Shewanella* promoter	This study
pGEN-*bla*$_{OXA\text{-}1408}$	Expression plasmid for the novel OXA-1408 variant under its native *Shewanella* promoter	This study
pGEN-*bla*$_{OXA\text{-}1410}$	Expression plasmid for the novel OXA-140 variant under its native *Shewanella* promoter	This study
pGEN-*bla*$_{OXA\text{-}1413}$	Expression plasmid for the novel OXA-140 variant under its native *Shewanella* promoter	This study
pGEN-*bla*$_{OXA\text{-}1417}$	Expression plasmid for the novel OXA-140 variant under its native *Shewanella* promoter	This study
**Primers**		
**Cloning**		
blaOXA551-KpnI-F	ATA<u>GGTACC</u>ttttgatttccctgtagtgattagacg	This study
blaOXA551-XmaI-R	TA<u>CCCGGG</u>ctaaggaataacgtgttccagt	This study
Bla551-585up-BamHI-F	ATA<u>GGATCC</u>ctgttgccataatctgtgacct	This study
Bla551-585up-PspOMI-R	TAATTT<u>GGGCCC</u>aaagtctccttgtagaaaaca	This study
Bla551-600Down-PspOMI-F	TAATTT<u>GGGCCC</u>cccatgagggattgcaggc	This study
blaOXA54-KpnI-F	ATA <u>GGTACC</u> tggcgtttcctttttattagt	This study
blaOXA54-XmaI-R	TA <u>CCCGGG</u> ctatggaattatcttttcctgt	This study
pGEN-Tc-XbaI-F	ATA<u>TCTAGA</u>tcggcaaggtgttctggtc	This study
pGEN-Tc-SpeI-R	TAT<u>ACTAGT</u>tcaggtcgaggtggcccg	This study
**Inspection of gene deletion**		
Check-bla551-F	cgtcaatatgacctgactgcac	This study
Check-bla551-R	gtaacttgagtctgacccaagtc	This study
**Sanger sequencing**		
Check-Tc-insert-F	TTCACTTATCTGACACGAAAATCGC	This study
Check-Tc-insert-R	AGCGGTATCATCAACAGGCT	This study
pGEN-MCS-Check-F	TCTCACTTCCCTGTTAAGTATCTCC	This study
pGEN-MCS-Check-R2	ACGGGGCTATCTTCTTTCTGC	This study
pKNG101-F2	CGACACTGAATACGGGGCAA	This study
pKNG101-R	ACATGAGAATTCCCCTGGATTTCA	This study

*Uppercase = Nucleotides introduced for restriction digestion or 5' overhangs for optimal DNA cleavage. Restriction sites are underlined.

SUPPLEMENTAL REFERENCES:

1. Ferrieres L, Hemery G, Nham T, Guerout AM, Mazel D, Beloin C, Ghigo JM. 2010. Silent mischief: bacteriophage Mu insertions contaminate products of Escherichia coli random mutagenesis performed using suicidal transposon delivery plasmids mobilized by broad-host-range RP4 conjugative machinery. J Bacteriol 192:6418-27.
2. Kaniga K, Delor I, Cornelis GR. 1991. A wide-host-range suicide vector for improving reverse genetics in gram-negative bacteria: inactivation of the blaA gene of Yersinia enterocolitica. Gene 109:137-41.
3. Lane MC, Alteri CJ, Smith SN, Mobley HL. 2007. Expression of flagella is coincident with uropathogenic Escherichia coli ascension to the upper urinary tract. Proc Natl Acad Sci U S A 104:16669-74.
